# Dietary Changes and Their Predictors in the First Year After Childbirth in Women with Gestational Diabetes Mellitus: A Post Hoc Longitudinal Analysis from the Face-it Trial

**DOI:** 10.1016/j.tjnut.2025.10.025

**Published:** 2025-10-18

**Authors:** Anju Rawal, Helle Terkildsen Maindal, Inger Katrine Dahl-Petersen, Dorte Møller Jensen, Peter Damm, Per Ovesen, Christina Anne Vinter, Elisabeth R Mathiesen, Ulla Kampmann, Dirk Lund Christensen, Karoline Kragelund Nielsen, Face-it Study Group

**Affiliations:** 1 Department of Prevention, Health Promotion & Community Care, Copenhagen University Hospital – Steno Diabetes Center Copenhagen, Herlev, Denmark; 2 Department of Public Health, Faculty of Health, Aarhus University, Aarhus, Denmark; 3 Steno Diabetes Center Odense, Odense, Denmark; 4 Department of Gynecology and Obstetrics, Odense University Hospital, Odense, Denmark; 5 Department of Clinical Research, Faculty of Health Sciences, University of Southern Denmark, Odense, Denmark; 6 Center for Pregnant Women with Diabetes, Department of Gynaecology, Fertility and Obstetrics, Copenhagen University Hospital – Rigshospitalet, Copenhagen, Denmark; 7 Department of Clinical Medicine, Faculty of Health and Medical Sciences, University of Copenhagen, Copenhagen, Denmark; 8 Steno Diabetes Center Aarhus, Aarhus, Denmark; 9 Department of Clinical Medicine, Faculty of Health, Aarhus University, Aarhus, Denmark; 10 Department of Gynecology and Obstetrics, Aarhus University Hospital, Aarhus, Denmark; 11 Center for Pregnant Women with Diabetes, Department of Endocrinology and Metabolism, Copenhagen University Hospital – Rigshospitalet, Copenhagen, Denmark; 12 Department of Public Health, University of Copenhagen, Copenhagen, Denmark

**Keywords:** dietary quality, gestational diabetes mellitus, lifestyle, maternal health, postpartum diet, diet predictors

## Abstract

**Background:**

Healthy diet is essential to reduce the increased risk of developing type 2 diabetes mellitus (T2DM) among women with previous gestational diabetes mellitus (GDM).

**Objectives:**

This study investigates dietary changes and predictors of dietary improvement during first year after childbirth among women with GDM.

**Methods:**

This post hoc longitudinal analysis used data from the Face-it randomized controlled trial, which evaluated a health promotion intervention for women with recent GDM. As the intervention had no effect on diet, data from both intervention and usual care groups were pooled, collected at baseline and follow-up (3 and 12 mo after childbirth). Dietary quality score (DQS) was used to assess self-reported dietary habits. Predictor variables included body mass index (BMI), risk perception of T2DM, self-perceived dietary habits, social support, breastfeeding status, and mental well-being. Paired t-test and ordinal logistic regression adjusted for randomization group were conducted.

**Results:**

This study included 232 women. The overall mean DQS did not change from baseline to follow-up; however, 66% women modified their dietary quality, with an equal split between improvement and decline. Higher odds of dietary improvement were seen in women with baseline BMI ≥30 kg/m^2^ [odds ratio (OR): 3.29; 95% confidence interval (CI): 1.60, 6.80] and BMI 25–29.9 kg/m^2^ (OR: 2.41; 95% CI: 1.28, 4.54) compared with those with a BMI <25 kg/m^2^. Women who perceived their diet as unhealthy had increased odds of improvement compared with those who perceived it as healthy (OR: 3.84; 95% CI: 1.40, 10.56). Fully breastfeeding women at baseline had lower odds of dietary improvement than nonbreastfeeding women (OR: 0.39; 95% CI: 0.18, 0.84). No associations were found for risk perception of T2DM, social support, and mental well-being.

**Conclusions:**

Dietary patterns after a GDM-affected pregnancy are heterogeneous, underscoring the importance of tailored dietary interventions addressing individual needs to improve dietary quality and reduce the risk of T2DM.

## Introduction

Gestational diabetes mellitus (GDM) is defined as glucose intolerance that develops or is detected for the first time during pregnancy [1]. In Denmark, the estimated prevalence of GDM is ∼6% [2]. GDM increases the risk of adverse pregnancy outcomes, such as macrosomia, preeclampsia, and preterm delivery, while also presenting long-term health implications for both mothers and offspring [3]. A recent systematic review and meta-analysis showed that women with a history of GDM have a 10-fold increased risk of developing type 2 diabetes mellitus (T2DM), with the largest relative risk within the first 5 y after GDM [4]. Additionally, early-onset T2DM, diagnosed before the age of 40, is associated with a 4-fold increased risk of mortality compared with the general population, with the risk rising further the younger the age at diagnosis [5].

Given these risks, implementing effective health interventions for women with a history of GDM is essential for promoting long-term health and reducing the likelihood of T2DM and cardiovascular morbidity and mortality [4,6]. Research indicates that making health behavior changes, such as adopting a healthy diet, can reduce the risk of diabetes by 40%–57% in this population [7,8]. Although dietary management is the primary treatment of GDM during pregnancy, healthy dietary changes may cease after childbirth, resulting in a decline in healthy eating habits [9]. Sustaining the dietary modifications made during pregnancy is crucial, and interventions aimed at promoting long-term adherence to a healthy diet could have significant health benefits for this population.

A Danish study conducted >2 decades ago found that although the majority of women with prior GDM worried about developing T2DM, only a few improved their diet and physical activity after pregnancy [10]. The study further reported that although high-fat diet intake decreased after childbirth compared with prepregnancy, these improvements were insufficient to prevent weight gain [10]. However, research remains limited on how this group’s overall dietary quality evolves after childbirth.

Moreover, little is known about the factors that predict dietary improvement in women with a history of GDM. Identifying these potentially modifiable factors is crucial for developing effective interventions. This study focuses on key predictors of dietary improvement. These include BMI (in kg/m^2^), self-perceived dietary habits, risk perception of T2DM, social support, breastfeeding, and mental health [[10], [11], [12], [13], [14], [15], [16], [17], [18], [19]]. BMI has been widely associated with dietary quality, with higher BMI linked to lower adherence to healthy diets [11,12]. Self-perceived dietary habits influence motivation, as individuals who recognize poor eating behaviors are more likely to seek improvements [[13], [14], [15]]. Risk perception of T2DM is a known psychological driver of preventive behaviors, as women with a heightened awareness of their risk may be more motivated to adopt dietary modifications [16,17]. Greater social support was associated with healthy dietary practices, such as increasing fruit and vegetable intake [18]. Breastfeeding has also been linked to healthier eating patterns, as women often associate their nutritional status with the quality of their breast milk [19]. Lastly, mental health is a strong predictor of dietary behavior, as poor mental health is often associated with a lower intake of fruits and vegetables and higher consumption of processed foods [20].

Despite these insights, few studies have comprehensively explored these factors, and a notable gap remains in research focused on this high-risk group of women with recent GDM. Therefore, this study aims to investigate changes in dietary quality among women with recent GDM from 3 to 12 mo after childbirth and identify predictors of dietary improvements.

## Methods

This article presents a post hoc longitudinal data analysis from the Face-it randomized controlled trial (RCT). The Face-it study design, intervention, and outcomes have been described elsewhere [21,22]. It was a 2-arm RCT evaluating the effectiveness of a health promotion intervention targeting women with GDM and their families in the first year after childbirth. The intervention comprised multiple components, including health visitor-led home visits, health technology, and cross-sectoral collaboration within the Danish healthcare system [21]. The intervention addressed overall health rather than targeting dietary improvement alone. The RCT was conducted between 2019 and 2023 [21]. The main aim of the Face-it study was to reduce the risk of T2DM and improve quality of life; however, although effects were documented on some cardiometabolic risk factors, no effect of the intervention was seen on dietary quality [21]. This study, therefore, explores potential changes in women’s dietary quality during the first year after childbirth, irrespective of their randomization allocation in the study. To investigate this, we conducted a new analysis, pooling data from intervention and usual care groups and employing a longitudinal study design.

### Ethical considerations

This study follows the ethical guidelines adopted in the Face-it study. The Face-it study was conducted in accordance with relevant guidelines and regulations such as the Declaration of Helsinki [22,23]. The Scientific Ethics Committee of the Capital Region and the Danish National Committee on Health Research Ethics granted ethical approval for the Face-it study (approval no. H-18056033) [22,23]. Informed consent was obtained from all study participants in the Face-it study [23].

### Study participants and recruitment

Participants were recruited from 3 university hospitals in different cities in Denmark: Rigshospitalet (Copenhagen), Aarhus University Hospital, and Odense University Hospital. Women diagnosed with GDM and between 24 and 40 wk of pregnancy were invited to participate if they could communicate in Danish and provide written consent, attended antenatal care at the recruiting hospitals and lived in collaborating municipalities. Women involved in other postpartum interventions, those diagnosed with overt diabetes at baseline, and those unable to understand the informed consent or study procedures were excluded from the study.

**FIGURE 1:**
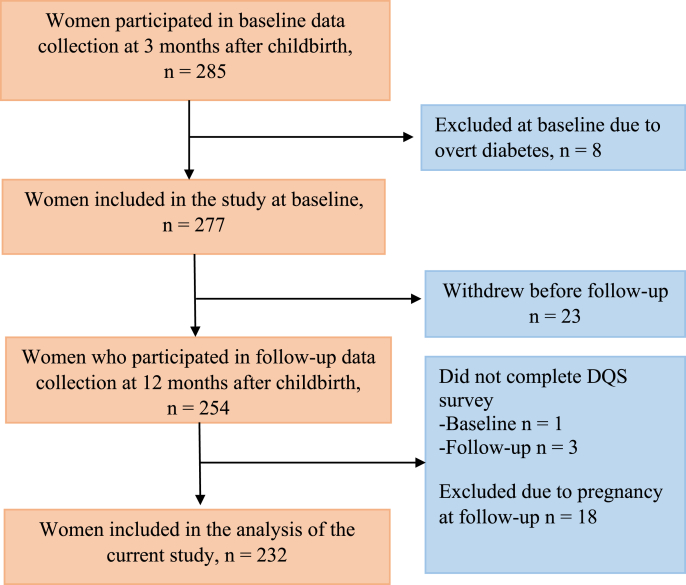
Flowchart of the study population. DQS, dietary quality score.

A total of 285 participated in the baseline visits conducted ∼3 mo after childbirth (Figure 1). Of these, 8 women were excluded due to overt diabetes diagnosed, resulting in 277 women at baseline. Additionally, 23 women withdrew before the follow-up, resulting in 254 women participating in the follow-up at 12 mo after childbirth. Furthermore, women who did not complete the dietary quality score (DQS) questionnaire (baseline *n* = 1, follow-up *n* = 3) and women who were pregnant during the follow-up visit (*n* = 18) were also excluded. This left 232 women included in the final analysis.

### Study measures

Data collection included self-reported electronic questionnaires and clinical assessments conducted by trained healthcare professionals. The questionnaires covered background characteristics, including marital/cohabitation status, educational attainment, income, and country of birth. Additionally, the questionnaires gathered data on health-related behaviors such as dietary quality, risk perception of T2DM, self-perceived dietary habits, social support, breastfeeding, mental well-being, and physical activity. Although the Face-it study collected data at baseline and follow-up, this study primarily utilized baseline data, except for dietary quality, which was analyzed from both baseline and follow-up assessments.

#### Outcome – dietary quality

The outcome of this study was the change in dietary quality from 3 to 12 mo after childbirth. Dietary quality was assessed using the DQS questionnaire from 2017, a validated 23-item questionnaire designed to evaluate diet in relation to cardiovascular disease risk and later validated for cardiometabolic risk [24,25]. The DQS is based on 4 main food groups: fat, fish, fruits, and vegetables. Higher DQS scores have been associated with lower adiposity (waist circumference, visceral fat, total fat mass, and percentage), which are important biomarkers of T2DM, as well as greater intake of fiber, whole grains, vitamins, and minerals, and lower intake of saturated fat [24,25].

The total DQS score was calculated by summing scores from each food group converted to a 1–9 scale, with higher scores indicating healthier dietary habits. DQS scores were further categorized into “Unhealthy” (1–3 score), “Average” (4–6 score), and “Healthy” (7–9 score) groups [24,25]. For further analysis, the change in the DQS score was determined by subtracting the baseline DQS score from the follow-up DQS score. On the basis of this change, participants were classified into 3 distinct DQS change categories: “Declined” if their DQS score decreased by ≥1 point, “No change” if there was no difference between scores, and “Improved” if their DQS score increased by ≥1 point.

#### Predictors

##### BMI

Baseline body weight and height were obtained during the physical examination by trained healthcare professionals. BMI values were calculated using the formula: weight (kg)/height (m^2^). On the basis of WHO BMI categories, participants were categorized as normal weight (BMI 18.5–24.9 kg/ m^2^), overweight (BMI 25–29.9), and obese (BMI ≥30) [26].

##### Risk perception for T2DM

Risk perception for T2DM was measured using a single-item question: “What do you think your risk or chance is of getting diabetes over the next 10 y?”[27]. Participants were presented with the following response options: “Almost no chance,” “Slight chance,” “Moderate chance,” and “High chance.” The responses were then categorized as follows: “Almost no chance” and “Slight chance” were categorized as low-risk perception, “Moderate chance” as moderate risk perception, and “High chance” as high-risk perception.

##### Self-perceived dietary habits

Participants were asked to evaluate their current dietary habits by responding to “How do you rate your overall eating habits?” using a 5-point Likert scale. The scale ranged from 1 “Very healthy” to 5 “Very unhealthy.” For the analysis, the responses “Very healthy” and “Healthy” were combined into a single category labeled “Healthy,” “Average” remained unchanged, whereas the responses “Unhealthy” and “Very unhealthy” were grouped as “Unhealthy.”

##### Social support

The perceived level of social support was assessed using the Social Support for Healthy Eating (SSHE) scale, which was adapted from Sallis et al. [28]. This scale comprises 2 subscales: encouragement and discouragement, each with 5 items that evaluate behaviors and comments from others regarding healthy eating. Participants rated each item on a 5-point Likert scale, answering the same questions separately for family and friends. This approach resulted in 4 subscale scores: Family Encouragement, Family Discouragement, Friends’ Encouragement, and Friends’ Discouragement. Each score ranges from 5 to 25, with higher scores in the encouragement subscale indicating greater social support for healthy eating, whereas higher scores in the discouragement subscale indicate lower support. The sums of the items from both the Encouragement and Discouragement subscales for family and friends were included as continuous variables in the analyses.

##### Breastfeeding

Breastfeeding status was assessed by asking, “What has your baby been fed in the last 7 days?” Response categories included: “only breastmilk,” “breastmilk supplemented with a breastmilk substitute once,” “breastmilk supplemented with a breastmilk substitute two or more times,” and “only breastmilk substitute.” In Denmark, full breastfeeding allows for the supplementation of water and/or a maximum of 1 meal of infant formula per week [29]. Partial breastfeeding is when, in addition to breast milk, the infant receives infant formula or other dietary elements more than once within a week or day [29]. Therefore, for this analysis, “only breastmilk” and “breastmilk supplemented with a breastmilk substitute once” were categorized as full breastfeeding, whereas “breastmilk supplemented with a breastmilk substitute two or more times” were classified as partial breastfeeding. The option “only breastmilk substitute” was categorized as no breastfeeding.

##### Mental well-being

Mental well-being was assessed using the WHO-5 Wellbeing Index, which consists of 5 statements that respondents rate on a scale from 0 to 5 [30]. The total raw score (0–25) was multiplied by 4 to produce a 0–100 scale. For analysis, scores were categorized as “poor” (<50) and “good” (≥50), based on established cut-offs indicating possible risk of mental health conditions [30].

#### Covariates

##### Sociodemographic characteristics

Age was extracted from participants’ civil registration numbers. Participants reported whether they had a partner (“Yes”/“No”). They also provided their highest completed level of education, categorized into 3 groups: 0–10 y (did not complete primary education), 11–14 y (completed ≤4 y of higher education), and >15 y (>4 y of higher education). Household income was assessed via self-reported total annual gross income (in Danish kroner, DKK), with options ranging from <200,000 DKK to over 1 million DKK, including “Do not know” and “Do not want to answer” options.

##### Physical activity

Physical activity levels were assessed using an adapted version of the International Physical Activity Questionnaire Short Form (IPAQ-SF) [31]. The IPAQ-SF assesses self-reported days and hours spent on physical activity during the last 7 d for 3 intensity domains: vigorous-intensity activities, moderate-intensity activities, and walking. On the basis of the responses obtained from the IPAQ, energy expenditure was calculated and expressed in metabolic equivalent of task (MET) min/wk [32,33]. MET-min/wk were calculated separately for each intensity level (vigorous = 8.0 × min × d; moderate = 4.0 × min × d; walking = 3.3 × min × d), and a total physical activity score was obtained by summing these values [32].

##### Randomization group

Adjusting for the randomization group was crucial in this study, as data from both intervention and control groups in the RCT were pooled. In the Face-it study, two-thirds of participants received the intervention, potentially affecting DQS scores. Although the intervention had no effect on DQS, accounting for randomization remained essential to isolate its potential influence on predictive variables [34].

### Statistical analyses

Statistical analysis was performed using the Statistical Package for the Social Sciences version 29.0.1.0 with a significance level set at *P* < 0.05. Continuous variables are presented as mean and SD. A paired t-test was used to assess whether there was a significant change in the DQS score from baseline to follow-up. To identify predictors of DQS score changes, ordinal logistic regression (OLR) was performed. Three models were constructed: *1*) a crude model with a single predictor, *2*) a multivariate model including all variables, and *3*) an adjusted model controlling for covariates. In this study, the “DQS change categories” were used as the dependent variable for OLR analysis, representing ordered categories of change scores. All assumptions for OLR were checked and met prior to analysis. Multicollinearity among predictors was assessed using variance inflation factors, and the proportional odds assumption was tested using the test of parallel lines.

## Results

### Background characteristics

The mean age of the participants was 32.7 y (± 4.5), and 92.7% reported having a partner. A total of 190 (82%) women had completed college or higher education, and 183 (79%) were born in Denmark. Other baseline characteristics and details of the predictive variables are presented in Table 1.

**TABLE 1:** Characteristics of study participants and predictive factors at baseline.

Total participants, *n*	232
Age (y), mean ± SD	32.7 ± 4.5
Have a partner, *n* (%)	215 (92.7)
Primiparous, *n* (%)	126 (54.3)
Education, *n* (%)	
0–10 y	42 (18.1)
11–14 y	111 (47.8)
≥15 y	79 (34.1)
Income in DKK, *n* (%)	
<200,000–500,000	69 (29.7)
500,000–8000,000	84 (36.2)
800,000 ≥ 1000,000	58 (25.0)
Do not know/do not want to answer	21 (9.1)
Country/region of birth, *n* (%)	
Denmark	183 (78.9)
Europe	18 (7.8)
East and Southeast Asia	14 (6.0)
South Asia	5 (2.2)
Others	12 (5.2)
Employment, *n* (%)	
Employed	172 (74.1)
Student	27 (11.6)
Unemployed	28 (9.1)
Long-term sick leave/outside the labor market	5 (2.1)
BMI (kg/m^2^), *n* (%)	
Mean ± SD	27.8 (5.4)
Normal weight (18.5–24.9)	78 (33.6)
Overweight (25–29.9)	87 (37.5)
Obesity (≥30)	67 (28.9)
Risk perception of T2DM, *n* (%)	
Low	135 (58.2)
Moderate	82 (35.3)
High	15 (6.5)
Self-perceived dietary habits, *n* (%)	
Healthy	54 (23.3)
Average	152 (65.5)
Unhealthy	26 (11.2)
Social support score (mean ± SD)	
Family encouragement	11.3 ± 4.88
Family discouragement	9.03 ± 3.35
Friends’ encouragement	8.51 ± 4.07
Friends’ discouragement	7.05 ± 2.82
Breastfeeding, *n* (%)	
Full	163 (70.3)
Partial	35 (15.1)
No	34 (14.7)
WHO-5 mental well-being	
Mean ± SD	61.7 (14.7)
Good (≥50)	184 (79.3)
Poor (<50)	48 (20.7)
Time from childbirth to baseline visit in weeks (mean ± SD)	11.9 ±2.4
Time from childbirth to follow-up visit in weeks (mean ± SD)	53.7 ± 3.3

Abbreviation: T2DM, type 2 diabetes mellitus; DKK, Danish kroner.

### Dietary quality at baseline and follow-up

Table 2 shows the consumption of various food groups and DQS scores at baseline and follow-up. At baseline, 20% of women had healthy dietary habits as assessed by DQS, and 12% were categorized as unhealthy. At the follow-up visit, the proportion in both groups had declined; 15% were categorized as having healthy dietary habits and 8% as having unhealthy dietary habits. Approximately 20% of women reported a low or negligible saturated fat intake at both baseline and follow-up. Fish consumption of <200 g/wk was reported by 67% of women at baseline and 65% at follow-up. The proportion of women consuming >3 pieces of fruit daily was 10% at baseline and 6% at follow-up. Approximately 30% consumed >5 servings of vegetables per week at both time points.

**TABLE 2:** Food group consumption and dietary quality score at baseline and follow-up.

Food group	Frequency	Baseline score *n* (%)	Follow-up score *n* (%)
Fat (saturated fat)	Mean ± SD	1.10 ± 0.58	1.22 ± 0.52
	Low (mostly olive oil, no other fat)	52 (22.4)	44 (19.0)
	Moderate (vegetable margarine and oil)	152 (65.5)	167 (72.0)
	High (margarine, butter, blended spread, lard)	28 (12.1)	21 (9.0)
Fish	Mean ± SD	0.91 ± 0.57	0.98 ± 0.6
	≥200 g/wk	28 (12.1)	39 (16.8)
	<200 g/wk	156 (67.2)	150 (64.7)
	No intake	48 (20.7)	43 (18.5)
Fruits	Mean ± SD	0.91 ± 0.53	0.98 ± 0.6
	≥3 pieces/d	23 (9.9)	14 (6.1)
	≥3 pieces//wk and ≤2 pieces/d	166 (71.6)	185 (79.7)
	≤2 pieces/wk	43 (18.5)	33 (14.2)
Vegetables	Mean ± SD	1.23 ± 0.6	1.22 ± 0.59
	≥5–7 servings/wk	74 (31.9)	70 (30.2)
	3–4 servings/wk	137 (59.1)	142 (61.2)
	≤2 servings/wk	21 (9.1)	20 (8.6)
Total DQS score	(mean ± SD)	5.16 ± 1.44	5.22 ± 1.24
DQS categories	Healthy dietary habits (7–9 points)	46 (19.8)	34 (14.7)
	Average dietary habits (4–6 points)	159 (68.5)	180 (77.6)
	Unhealthy dietary habits (1–3 points)	27 (11.6)	18 (7.8)

Abbreviation: DQS, dietary quality score.

### Change in dietary quality from baseline to follow-up

There was no statistically significant difference in the mean DQS score of all participants between baseline (5.16 ± 1.44) and follow-up (5.22 ± 1.24), [t (231) = 0.695; *P* = 0.488]. Similarly, no significant changes were observed in the consumption of individual food groups, including fat, fish, fruits, and vegetables, during this period. However, at the individual level, 66% of women had a change in DQS score, with 33% improving and 33% declining from baseline to follow-up.

When examining shifts in DQS categories, 16% of women moved to a healthier category, transitioning from “Unhealthy” to “Average” or “Average” to “Healthy.” Conversely, 17% moved to an unhealthier category, shifting from “Healthy” to “Average” or “Average” to “Unhealthy.” No participants moved directly from “Unhealthy” to “Healthy” or from “Healthy” to “Unhealthy.”

Most women who improved their DQS score were from the average dietary habits group at baseline (52/76) (Table 3). The highest proportion of improvement, however, was in the unhealthy group, where 81.5% (22/27) improved, compared with 32.7% (52/159) in the average group and 4.3% (2/46) in the healthy group. Conversely, among women with initially healthy dietary habits, 73.9% (34/46) decreased in their DQS score.

**TABLE 3:** Change in dietary quality score from baseline to follow-up by dietary habits at baseline.

Change in DQS score	Healthy dietary habits (7–9 points) (*n* = 46)	Average dietary habits (4–6 points) (*n* = 159)	Unhealthy dietary habits (1–3 points) (*n* = 27)
Decline, *n* (%)	34 (73.9)	44 (27.7)	0 (0.0)
No change, *n* (%)	10 (21.7)	63 (39.6)	5 (18.5)
Improve, *n* (%)	2 (4.3)	52 (32.7)	22 (81.5)

Dietary habits at baseline are categorized based on DQS: Healthy (7–9), Average (4–6), Unhealthy (1–3). Change in DQS from baseline to follow-up is assessed as a continuous variable. Participants in the “Healthy” group may still show improvement if their initial score was below the maximum (e.g., from 7 to 9).

Abbreviation: DQS, dietary quality score.

### Predictors of dietary improvement

In the adjusted OLR analysis, several factors were identified as significant predictors of dietary improvement based on DQS change categories (decline, no change, and improved) (Table 4). Women with a BMI ≥30 at baseline had significantly higher odds of dietary improvement compared with those with a BMI <25 [odds ratio (OR): 3.29; 95% confidence interval (CI): 1.60, 6.80; *P* = 0.001] at follow-up. Similarly, those with a BMI of 25.0–29.9 had odds of 2.41 (95% CI: 1.28, 4.54; *P* = 0.006). Individuals who perceived their diet as unhealthy had higher odds of dietary improvement than those who perceived it as healthy (OR: 3.84; 95% CI: 1.40, 10.56; *P* = 0.009). Fully breastfeeding women at baseline had lower odds of dietary improvement than those not breastfeeding (OR: 0.39; 95% CI: 0.18, 0.84; *P* = 0.016). Other factors, including perceived risk of T2DM, social support, and mental well-being, were not significant predictors of dietary improvement.

**TABLE 4:** Baseline variables predicting improvement in dietary quality score (*n* = 232).

Variables	Crude		Model 1		Model 2	
	OR (95% CI)	*P* value	OR (95% CI)	*P* value	OR (95% CI)	*P* value
BMI (kg/m^2^)						
Obesity (≥30.0)	2.44 (1.33, 4.51)	0.004	2.90 (1.47, 5.71)	0.002	3.29 (1.60, 6.80)	0.001
Overweight (25.0–29.9)	1.78 (1.01, 3.14)	0.047	2.18 (1.19, 4.03)	0.012	2.41 (1.28, 4.54)	0.006
Normal weight (18.5–24.9)	1		1		1	
Risk perception for T2DM						
High	1.52 (0.56, 4.14)	0.408	0.70 (0.25, 2.03)	0.516	0.59 (0.20, 1.74)	0.341
Moderate	2.62 (0.93, 7.38)	0.067	1.62 (1.00, 2.87)	0.097	1.58 (0.88, 2.83)	0.126
Low	1		1		1	
Self-perceived dietary habits						
Unhealthy	3.59 (1.45, 8.86)	0.006	3.34 (1.25, 8.95)	0.016	3.84 (1.40, 10.56)	0.009
Average	1.14 (0.65, 2.03)	0.647	0.98 (0.53, 1.80)	0.946	1.02 (0.54, 1.03)	0.939
Healthy	1		1		1	
Social support						
Family encouragement	0.98 (0.93, 1.03)	0.379	0.96 (0.90, 1.03)	0.259	0.96 (0.90, 1.03)	0.296
Family discouragement	1.01 (0.94, 1.08)	0.851	1.04 (0.95, 1.13)	0.396	1.04 (0.95, 1.13)	0.377
Friends’ encouragement	0.96 (0.91, 1.02)	0.225	0.95 (0.87, 1.04)	0.238	0.96 (0.87, 1.05)	0.338
Friends’ discouragement	0.96 (0.88, 1.04)	0.340	0.96 (0.86, 1.08)	0.496	0.95 (0.85, 1.06)	0.371
Breastfeeding						
Full	0.44(0.22, 0.87)	0.019	0.42 (0.20, 0.89)	0.023	0.39 (0.18, 0.84)	0.016
Partial	0.49 (0.20, 1.18)	0.111	0.42 (0.16, 1.06)	0.066	0.39 (0.15, 1.00)	0.051
No	1		1		1	
WHO-5 well-being						
Good (≥50)	0.98 (0.54, 1.75)	0.934	1.13 (0.61, 2.10)	0.692	1.25 (0.66, 2.35)	0.499
Poor (<50)	1		1		1	

The table represents the ordinal logistic regression analysis used to predict improvements in dietary quality score (DQS) from baseline variables.

Crude: unadjusted analysis including a single predictor variable.

Model 1: multivariate analysis with all predicting variables included.

Model 2: adjusted for randomization group, age, income, education, and physical activity.

Abbreviations: CI, confidence interval; OR, odd ratio; T2DM, type 2 diabetes.

## Discussion

### Change in dietary quality

This study examined changes in dietary quality during the first year after childbirth and identified predictors of dietary improvement among women with recent GDM. The results show that one-third of participants experienced an improvement in their DQS score, one-third experienced a decline, and one-third showed no change from baseline to follow-up. The result also suggests that few women had healthy dietary habits, with only 20% classified as having a healthy diet at baseline and 12% at follow-up.

The DQS was developed based on the Danish food-based dietary guidelines (FBDG) and reflects adherence to national dietary recommendations [35]. The low DQS scores in this cohort indicate poor adherence to the FBDG. Around 65% of women consumed <200 g of fish per week (compared with 350 g guideline), fewer than 10% ate 3 pieces of fruit daily, and only ∼30% reached 5 servings of vegetables per week, all well below the recommended 6 pieces of fruit and vegetables per day [36]. Similar findings were reported from a previous study in which women did not meet national dietary guidelines for many food groups, such as dairy intake, fruits, and vegetables [37]. These findings are concerning given the well-established relationship between diet and cardiometabolic health. Previous studies have shown that higher DQS and adherence to Danish FBDG are inversely associated with cardiometabolic risk profiles, such as low-density lipoprotein-cholesterol, waist circumference, visceral fat, total fat mass, and a positive association with high-density lipoprotein-cholesterol [25,38]. These biomarkers are critical for long-term metabolic health, particularly in women with prior GDM who are at elevated risk of T2DM.

To our knowledge, this is the first prospective study on dietary transitions and their predictors among women with recent GDM, with prior research primarily qualitative and showing similar patterns. For instance, a French qualitative study, conducted 6–12 mo after childbirth, revealed that women reported dietary changes ranging from meal frequency to balanced diets [39]. Another qualitative study from Singapore reported excessive consumption of refined carbohydrates, such as white rice, white bread, pasta, crackers, pastries, and sweetened beverages, alongside a low intake of fruits and vegetables [40]. These findings highlight that dietary behaviors after GDM pregnancies are dynamic and vary substantially between individuals.

Although our study identified equal proportions of women with improved and declined DQS scores, it cannot be assumed that all women with improvements were following a completely healthy diet, nor that all those with declines were eating poorly. For example, some women may have shown only modest improvements, for example DQS increasing from 2 to 3, whereas others achieved substantially higher scores, such as 9. Nonetheless, even minor improvements in dietary quality can have meaningful health implications and reduce the risk of T2DM, whereas small declines may increase the T2DM risk, particularly for this high-risk group [41,42]. Only one-third of women in our study improved their diet quality, highlighting a key gap and suggesting that many may face challenges in improving their diet during this time, potentially placing them at an even higher risk for T2DM.

### Predictor of dietary improvement

Our study identified several predictors of dietary improvement, with higher BMI emerging as one of the key positive factors. Women with BMI ≥30 had over 3 times the odds, whereas those with BMI of 25.0–29.9 had more than twice the odds of dietary improvement compared with women with a BMI <25. A previous study similarly reported reductions in high-fat diet among women with a BMI >25 after childbirth compared with prepregnancy diets [10]. Several factors may explain this association: overweight and obese women with prior GDM often have a heightened perception of their risk for developing T2DM, which may motivate them to lose weight by adopting a healthier diet [43,44]. In addition, healthcare professionals may be more likely to provide dietary counseling and emphasize weight management strategies to women with higher BMI, reinforcing the need for dietary improvements [45].

Women who perceived their diets as unhealthy had nearly 4 times the odds of improving their dietary quality compared with those who viewed their diets as healthy. This finding highlights the importance of self-perception in driving dietary behavior change. Health behavior theories suggest that individuals are more likely to modify their behavior when they recognize a problem or perceive a gap between their current and ideal state [46]. It is also possible that women who perceived their diets as unhealthy had objectively poorer diets at baseline, as previous studies show that dietary self-perceptions often align with actual eating behaviors [47,48]. This self-realization may motivate women to adopt a healthy diet over time. Thus, helping women better understand their eating habits could be an effective strategy to encourage healthier dietary changes.

In contrast, breastfeeding emerged as a negative predictor; fully breastfeeding women had 61% lower odds of improving their dietary quality than those who did not breastfeed. One possible explanation is that breastfeeding mothers may already adhere to relatively healthier diets during this period, leaving less room or perceived need for further improvement. Breastfeeding is often accompanied by heightened motivation to maintain a nutritious diet due to its perceived importance for infant health and development [19]. This motivation may be even stronger among women with a history of GDM, as many were highly concerned about their infant’s health during pregnancy and may carry this concern after childbirth [49]. However, this motivation may diminish by 12 mo after childbirth as breastfeeding frequency decreases or ceases, and the infant transitions to solid food [50], which may lead mothers to place less emphasis on their own diet. Additionally, practical challenges such as returning to work, managing child feeding, and balancing infant care demands may further limit mothers’ capacity to prioritize or implement dietary improvements for themselves.

Neither risk perception of T2DM nor social support predicted dietary improvements in our study. Although previous research has linked lower risk perception to poorer dietary habits and greater social support to better diet quality, these factors did not predict dietary improvement over time in our study [16,51]. For example, a cross-sectional study in Australia found that greater risk perception was associated with reduced fruit and vegetable intake. However, this association became nonsignificant after adjusting for income [16], suggesting that factors such as socioeconomic status and access to resources may play a more substantial role than risk perception alone [52]. Higher risk perception motivates women with prior GDM to engage in lifestyle changes, including dietary improvements [53]. Nevertheless, a gap often exists between risk perception and behavior, as external barriers such as limited resources, time constraints, and competing family demands can impede their ability to act on this motivation [52]. This highlights the complex interplay between risk perception and socioeconomic factors in shaping dietary behaviors.

Similarly, our study focused only on social support from family and friends, but previous research suggests that women with recent GDM often seek support from the healthcare system to help manage their dietary needs alongside busy postpartum lifestyles [54]. Many report receiving little to no information about dietary guidelines after delivery, in contrast to the more intensive support provided during pregnancy [54]. Furthermore, a qualitative study among Danish women with prior GDM emphasized the critical role of partner support in sustaining healthy dietary habits [55]. Although 93% of our participants reported having a partner, the SSHE scale did not separate partners from other family members, potentially obscuring the unique impact of partner support. Given partners’ central role in household food choices, support from friends and other family members may exert comparatively less influence on dietary behavior. These findings underscore the need to differentiate the sources of social support, as assistance from partners and healthcare providers may be more influential in promoting a healthy diet during this period.

Finally, mental well-being did not predict dietary changes in our study, despite evidence from the general population suggesting a bidirectional relationship between diet and mental health [56]. No studies have specifically examined this relationship in women with prior GDM. The lack of a significant relationship in our findings may be due to postpartum dietary decisions being driven more by practical constraints such as time availability, childcare responsibilities, and breastfeeding demand rather than mental health status. Mental well-being may influence dietary behaviors in some populations, and pre-established dietary routines and childcare actors may substantially impact dietary improvements in this group.

### Strengths and limitations of the study

To our knowledge, this is the first prospective study to explore dietary changes and their predictors, specifically among women with recent GDM. By addressing this relatively under-researched area, the study provides valuable insights into the dynamics of dietary behavior during a critical period for this high-risk group. Another key strength of this study is its prospective longitudinal design, which allows for tracking women’s dietary intake over time, providing insights into changes in their dietary habits during the first year after childbirth. This approach reveals trends and patterns in food intake that might be missed in cross-sectional studies. Furthermore, data collection was rigorous: BMI was objectively measured by trained healthcare professionals, adding credibility to the findings. Validated tools like DQS and SSHE assessed dietary quality and social support, ensuring robust and reliable measurements.

One limitation of this study is the post hoc analysis of RCT data, where two-thirds of participants received a health promotion intervention. This may limit the generalizability of the findings to women with prior GDM who did not receive similar interventions despite adjustments for randomization group. Additionally, the study population may not be representative of the broader population, as participants in the Face-it study differed from nonparticipants, who had a higher prepregnancy BMI, lower rates of primiparity, and more multiple pregnancies [57]. Furthermore, women who chose to participate may have been more motivated to adopt behavioral changes compared with nonparticipants, potentially leading to a healthier dietary profile than what would be observed in the general population with prior GDM. Another limitation is the reliance on the DQS and IPAQ to assess dietary quality and physical activity, which may introduce recall bias. Moreover, the DQS includes only 4 food groups: fat, fish, fruits, and vegetables, and therefore does not capture potential changes in other dietary components such as legumes, dairy, and sugar-sweetened beverages. These limitations underscore the need for caution in interpreting our findings and highlight the importance of using more comprehensive and objective measures of dietary quality and physical activity in future research to enhance accuracy.

### Future implication

Given the low proportion of women with healthy dietary habits, dietary interventions should be universally offered to women with prior GDM. These should be incorporated into routine care through the healthcare system, similar to the dietary guidance provided during pregnancy. However, these interventions must be tailored, as a one-size-fits-all approach may not be effective due to the substantial individual variability in dietary changes. Women who have already improved their diets may require ongoing support to sustain changes or achieve further progress, whereas those whose dietary quality remained unchanged may benefit from additional guidance to promote positive modifications. Particular attention should be directed toward women whose dietary quality has declined, as maintaining a healthy diet is essential for reducing the risk of T2DM in this population. Additionally, interventions should specifically address groups identified in this study as less likely to improve their diets, including women with a BMI <25, those who perceive their diets as already healthy, and those who were breastfeeding. Targeted strategies should aim to optimize dietary habits in these groups, ensuring they receive appropriate support to maintain or enhance their nutritional intake.

Future studies should extend beyond the first year after delivery to assess long-term dietary variations, providing a broader overview of dietary patterns over time and help inform interventions. Although this study identified key predictors of dietary variation, additional factors may influence postpartum dietary behaviors. Further research should explore broader psychosocial and environmental determinants, such as social norms, cultural beliefs, healthcare accessibility, and health literacy, to provide a more comprehensive understanding of dietary patterns among women with a history of GDM. Investigating these influences could inform more targeted and sustainable dietary interventions, ultimately contributing to long-term T2DM prevention in this high-risk population.

## Conclusion

The study investigated changes in dietary quality among women with prior GDM and found that dietary quality did not change at the group level from 3 to 12 mo after childbirth. Although approximately one-third of women improved their dietary quality, an equal proportion showed no change, and another third experienced a decline, underscoring the complexity of promoting healthy dietary behaviors in this high-risk group. Furthermore, our investigation identified key predictors of dietary change. A higher BMI (≥25), the perception of having an unhealthy diet, and not breastfeeding at baseline were all associated with positive dietary changes at follow-up. These findings underscore the importance of developing tailored dietary interventions that address individual needs to improve dietary quality and reduce the risk of T2DM.<END ARTICLE>

## Author contributions

The authors’ responsibilities were as follows – AR, KKN: conceived the study; AR: prepared the manuscript under the supervision of HTM and KKN; HTM, KKN, DLC, AR: contributed to the analysis plan; AR: conducted the data analyses; HTM, IKD-P, DMJ, PD, PO, CAV, ERM, UK, KKN: contributed to the Face-it trial; and all authors: critically reviewed the manuscript and approved the final version.

## Data availability

No data are available. Data from the Face-it trial are currently being analyzed. The data generated and analyzed during this study are therefore not publicly available. Please contact the corresponding author in case of any questions regarding the data used for this study.

## Declaration of generative AI and AI-assisted technologies in the writing process

During the preparation of this work, the author (AR) used Grammarly to check for grammatical errors and to ensure the application of academic grammar. After using Grammarly, the author reviewed and edited the content as necessary and accepts full responsibility for the publication's content.

## Funding

The Face-it RCT is funded by an unrestricted grant from the Novo Nordisk Foundation (NNF17OC0027826). The funder had no role in the study design, data collection, analysis, interpretation, manuscript preparation, or publication of results.

## Conflict of interest

KKN, IKD-P, UK, PO, CAV, DMJ, HTM are/were employed full-time or part-time at the Steno Diabetes Centers in Copenhagen, Odense, or Aarhus. These centers are regional public hospitals and research institutions partially funded by grants from the Novo Nordisk Foundation. ERM reports a relationship with Novo Nordisk AS that includes, funding grants, speaking and lecture fees, advisory board, and travel reimbursement and has contracts with Novo Nordisk for the Expect Trial and the Evolve Trial investigating newer insulin analogs and insulin pump treatment in pregnant women with diabetes. PD has participated in clinical studies on the use of insulin in pregnant women with pre-existing diabetes in collaboration with Novo Nordisk AS, but no personal honorarium was involved. AR and DLC declare no conflicts of interest.
